# Supplementary material for: Procedures in Fecal Microbiota Transplantation for Treating Irritable Bowel Syndrome: Systematic Review and Meta-Analysis
Source: J Clin Med. 2023 Feb 21;12(5):1725. doi: 10.3390/jcm12051725 (PMC10003588; doi:10.3390/jcm12051725)
Supplement: Supplementary file 1 [file jcm-12-01725-s001.zip › jcm-2191679-supplementary.pdf]

SUPPLEMENTARY MATERIAL

Supplementary Table S1. The Rome IV criteria for IBS.

1. Recurrent abdominal pain, on average, at least 1 day per week in the last 3 months and associated with two or more or the following:

a. Related to defecation.
b. Associated with a change in frequency of stool;
c. Associated with a change in form of stool.

AND

2. Criteria fulfilled for the last 3 months with symptom onset at least 6 months before diagnosis:

IBS-C	IBS-D	IBS-M	IBS-U
≥25% of bowel movements of Bristol stool form types 1 or 2, and <25% of Bristol stool form types 6 or 7.	≥25% of bowel movements of Bristol stool form types 6 or 7, and <25% of Bristol stool form types 1 or 2.	≥25% of bowel movements of Bristol stool form types 1 or 2, and ≥25% of bowel movements of Bristol stool form types 6 or 7.	Patients who meet criteria for IBS, but who do not fall into one of the other three subgroups according to Bristol stool form type.

Abbreviations: IBS-C: IBS with constipation; IBS-D: IBS with diarrhea; IBS-M: IBS with mixed bowel habits; IBS-U: IBS unclassified.
Adapted from British Society of Gastroenterology guidelines on the management of irritable bowel syndrome. Gut 2021;70:1214-1240.10

Supplementary Table S2. Bristol stool form scale

Type 1	Separate hard lumps, like nuts (hard to pass).
Type 2	Sausage-shaped but lumpy.
Type 3	Like a sausage but with cracks on its surface.
Type 4	Like a sausage or snake, smooth and soft.
Type 5	Soft blobs with clear-cut edges (passed easily).
Type 6	Fluffy pieces with ragged edges, a mushy stool.
Type 7	Watery, no solid pieces.

Adapted from Validity and reliability of the Bristol Stool Form Scale in healthy adults and patients with diarrhea-predominant irritable bowel syndrome. Aliment Pharmacol Ther. 2016;44(7):693-703.

Supplementary Table S3. Query definition referring to fecal microbiota transplantation combined with terms referring to irritable bowel syndrome (#1 AND #2 AND #3).

#	PUBMED	SCOPUS	WEB OF SCIENCE	COCHRANE
**1**	"fecal"[Title/Abstract] OR "faecal"[Title/Abstract] OR "feces"[MeSH Terms] OR "feces"[Title/Abstract] OR "faeces"[Title/Abstract] OR "stool"[Title/Abstract] OR "gut"[Title/Abstract] OR "microbiota"[MeSH Terms] OR "microb*"[Title/Abstract] OR "microflora"[Title/Abstract]	TITLE-ABS-KEY(fecal) OR TITLE-ABS-KEY(faecal) OR TITLE-ABS-KEY(feces) OR TITLE-ABS-KEY(faeces) OR TITLE-ABS-KEY(stool) OR TITLE-ABS-KEY(gut) OR TITLE-ABS-KEY(microb*) OR TITLE-ABS-KEY(microflora)	TI=(fecal OR faecal OR feces OR faeces OR stool OR gut OR microb* OR microflora) OR AB=(fecal OR faecal OR feces OR faeces OR stool OR gut OR microb* OR microflora)	(fecal):ti,ab,kw OR (faecal):ti,ab,kw OR (feces):ti,ab,kw OR MeSH descriptor:[feces] explode all trees OR (stool):ti,ab,kw OR (gut):ti,ab,kw OR (microb*):ti,ab,kw OR MeSH descriptor: [microbiota] explode all trees OR (microflora):ti,ab,kw
**2**	"transplantation"[MeSH Terms] OR "transplant*"[Title/Abstract] OR "transfusion"[Title/Abstract] OR "implant*"[Title/Abstract] OR "donor*"[Title/Abstract] OR "enema"[Title/Abstract] OR "infusion"[Title/Abstract] OR "reconstitution"[Title/Abstract] OR "transfer"[Title/Abstract] OR]	TITLE-ABS-KEY(transplant*) OR TITLE-ABS-KEY(transfusion) OR TITLE-ABS-KEY(implant) OR TITLE-ABS-KEY(donor*) OR TITLE-ABS-KEY(enema) OR TITLE-ABS-KEY(infusion) OR TITLE-ABS-KEY(reconstitution) OR TITLE-ABS-KEY(transfer)	TI=(transplant* OR transfusion* OR implant OR donor* OR enema OR infusion OR reconstitution OR transfer OR bacteriotherapy) OR AB=(transplant* OR transfusion* OR implant OR donor* OR enema OR infusion OR reconstitution OR transfer)	MeSH descriptor: [transplantation] in all MeSH products OR (transplant*):ti,ab,kw OR (transfusion):ti,ab,kw OR (implant*):ti,ab,kw OR (donor*):ti,ab,kw OR (enema):ti,ab,kw OR (infusion):ti,ab,kw OR (reconstitution):ti,ab,kw OR (transfer):ti,ab,kw
**3**	"irritable bowel syndrome"[MeSH Terms] OR "irritable bowel syndrome"[Title/Abstract] OR "irritable colon"[Title/Abstract] OR "IBS"[Title/Abstract] OR "dysbiosis"[Title/Abstract] OR "quality of life"[Title/Abstract] OR "functional gastrointestinal disorders"[Title/Abstract] OR "functional gut disorders"[Title/Abstract] OR "functional bowel disorders"[Title/Abstract]	TITLE-ABS-KEY("irritable bowel syndrome") OR TITLE-ABS-KEY(irritable colon) OR TITLE-ABS-KEY("quality of life") OR TITLE-ABS-KEY("functional gastrointestinal disorders") OR TITLE-ABS-KEY("functional gut disorders") OR TITLE-ABS-KEY("functional bowel disorders")	TI=("irritable bowel syndrome" OR "irritable colon" OR dysbiosis OR "quality of life" OR "ibs severity scoring system" OR "functional gastrointestinal disorders" OR "functional gut disorders" OR "functional bowel disorders") OR AB=("irritable bowel syndrome" OR "irritable colon" OR "quality of life" OR "functional gastrointestinal disorders" OR "functional gut disorders" OR "functional bowel disorders")	MeSH descriptor: [irritable bowel syndrome] explode all trees OR (irritable bowel syndrome):ti,ab,kw OR (irritable colon):ti,ab,kw OR (IBS):ti,ab,kw OR MeSH descriptor: [dysbiosis] explode all trees OR (dysbiosis):ti,ab,kw OR (quality of life):ti,ab,kw OR (functional gastrointestinal disorders):ti,ab,kw OR (functional gut disorders):ti,ab,kw OR (functional bowel disorders):ti,ab,kw

FIGURES

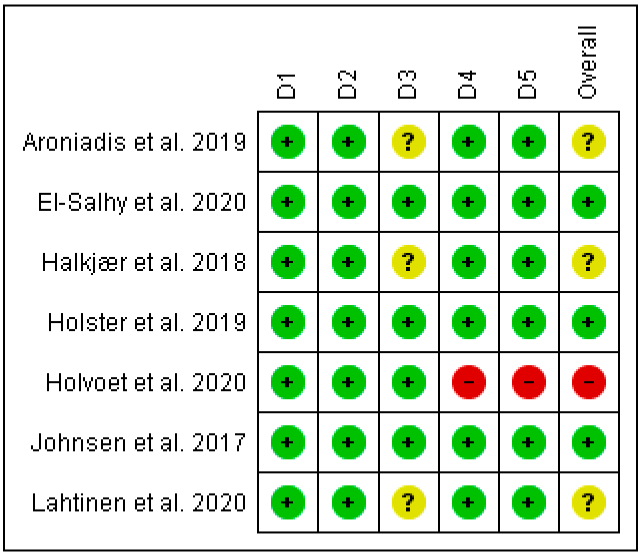

Domains
D1: Bias arising from the randomisation process
D2: Bias due to deviations from the intended interventions
D3: Bias due to missing outcome data
D4: Bias in measurement of the outcome
D5: Bias in selection of the reported result

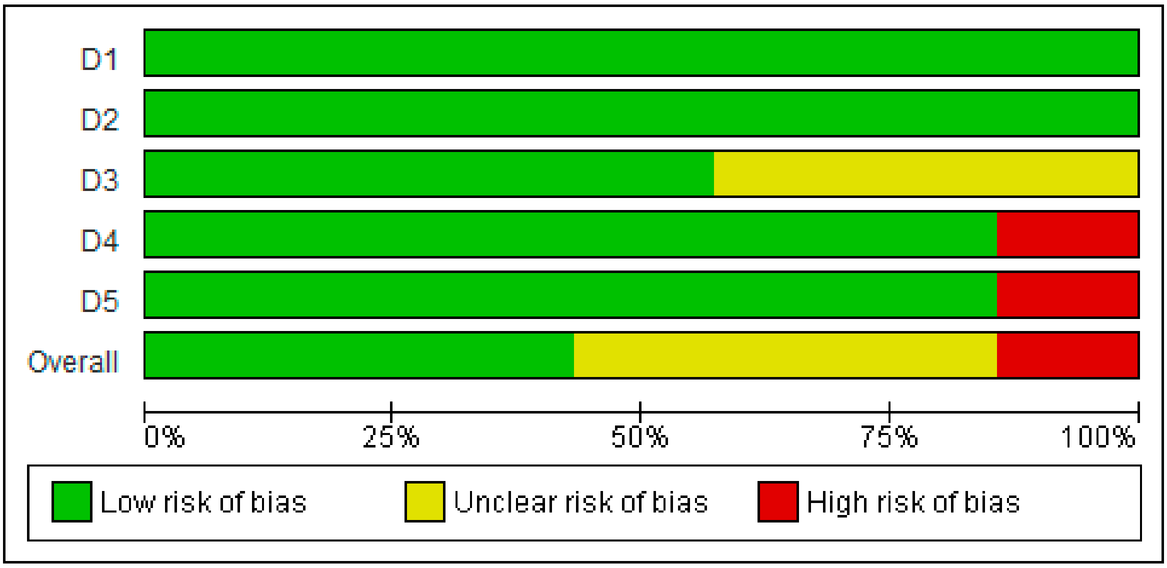

Supplementary Figure S1. Risk of bias: judgements about each risk of bias domain presented as percentages across all included studies.

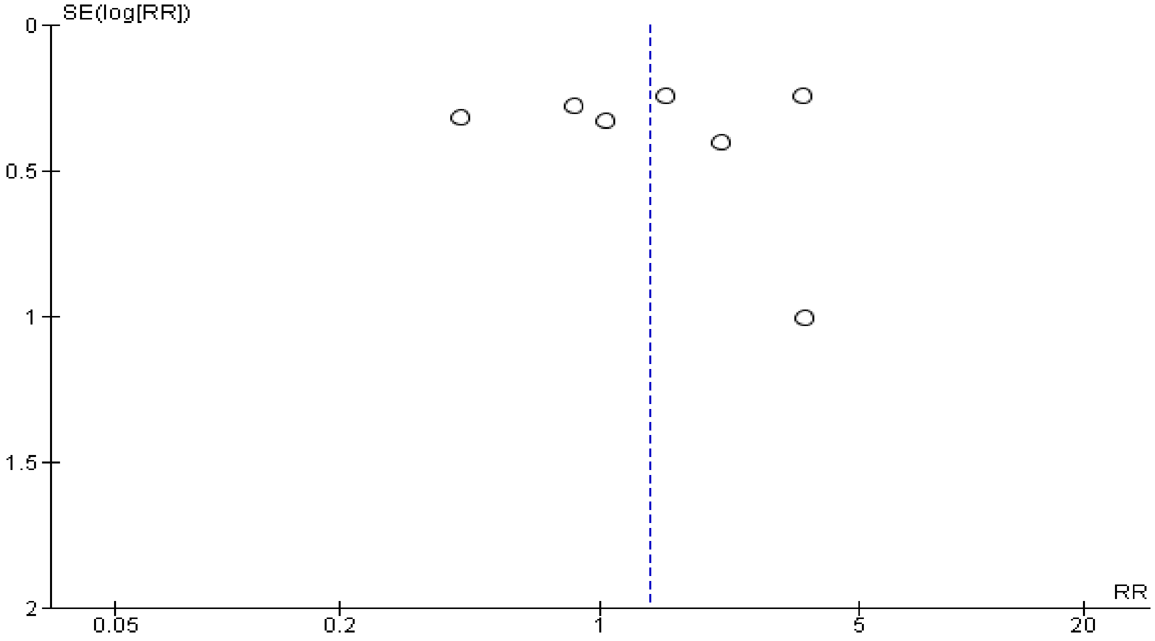

Supplementary Figure S2. Publication bias plot analysis. The RR of FMT responders is plotted on the x axis and the SE of the RR is plotted on the y axis. The vertical dotted line denotes the mean value of the RRs reported by the 7 included studies. Abbreviations: FMT: fecal microbiota transplantation; RR: risk ratio; SE: standard error.

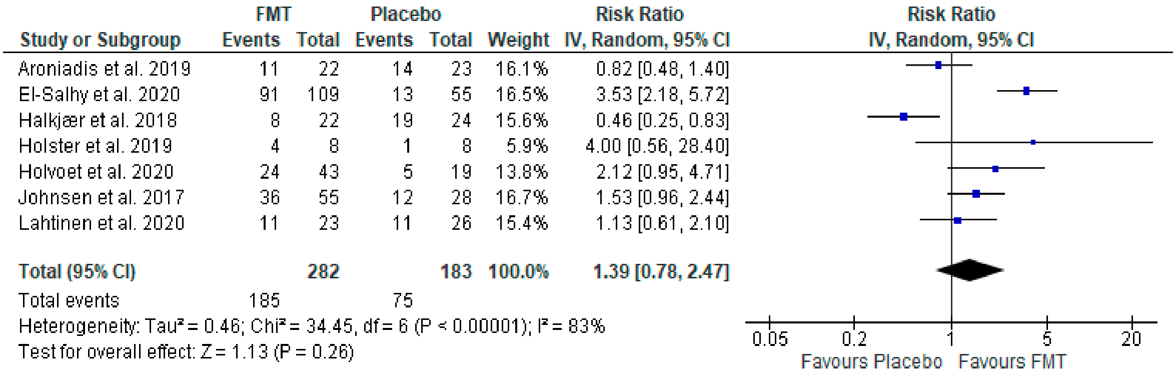

Abbreviations: CI: confidence interval; FMT: fecal microbiota transplantation; IBS: irritable bowel syndrome; RR: risk ratio.

Supplementary Figure S3. Forest plot of all studies for efficacy of FMT vs placebo on global improvement of IBS symptoms (without *intention-to-treat analysis*).
